# Krill Oil Supplementation Does Not Change Waist Circumference and Sagittal Abdominal Diameter in Overweight Women: A Pilot Balanced, Double-Blind, and Placebo-Controlled Clinical Trial

**DOI:** 10.3390/ijerph192013574

**Published:** 2022-10-20

**Authors:** Patrícia C. B. Lobo, Letícia N. Roriz, Jéssika M. Siqueira, Bruna M. Giglio, Ana C. B. Marini, Flávia C. Corgosinho, Raquel M. Schincaglia, Gustavo D. Pimentel

**Affiliations:** Faculty of Nutrition, Federal University of Goias, Goiânia 74605-080, Brazil

**Keywords:** krill oil, anthropometry, food intake, adiposity

## Abstract

An excess of body fat is one of the biggest public health concerns in the world, due to its relationship with the emergence of other health problems. Evidence suggests that supplementation with long-chain polyunsaturated fatty acids (omega-3) promotes increased lipolysis and the reduction of body mass. Likewise, this clinical trial aimed to evaluate the effects of supplementation with krill oil on waist circumference and sagittal abdominal diameter in overweight women. This pilot, balanced, double-blind, and placebo-controlled study was carried out with 26 women between 20 and 59 years old, with a body mass index >25 kg/m^2^. The participants were divided into the control (CG) (*n* = 15, 3 g/daily of mineral oil) and krill oil (GK) (*n* = 16, 3 g/daily of krill oil) groups, and received the supplementation for eight weeks. Food intake variables were obtained using a 24 h food recall. Anthropometric measurements (body mass, body mass index, waist circumference, and sagittal abdominal diameter) and handgrip strength were obtained. After the intervention, no changes were found for the anthropometric and handgrip strength variables (*p* > 0.05). Regarding food intake, differences were found for carbohydrate (*p* = 0.040) and polyunsaturated (*p* = 0.006) fatty acids, with a reduction in the control group and an increase in krill oil. In conclusion, supplementation with krill oil did not reduce the waist circumference and sagittal abdominal diameter. Therefore, more long-term studies with a larger sample size are necessary to evaluate the possible benefits of krill oil supplementation in overweight women.

## 1. Introduction

Obesity has a multifactorial etiology and is directly associated with emotional, behavioral, physiological, environmental, and genetic factors. In the last years, mainly due to the nutritional transition and the modern environment, its prevalence has been increasing and almost tripled between 1975 and 2016 [1]. The overweight treatment consists of changing lifestyle, which involves changes in eating habits, controlling body mass, practicing physical activity, and combating smoking [2]. Corroborating with that, it is suggested that supplementation with omega-3 long-chain polyunsaturated fatty acids (n-3 PUFA) may suppress the activity of the malonyl-CoA enzyme, favoring the beta-oxidation and the activity of lipolytic enzymes, increasing lipolysis and leading to a greater loss of body fat [3].

In this context, *Euphasia superba* (Antarctic krill), the main source of commercially available krill oil, is one of the most important marine species in Antarctica, which has a high content of n-3 PUFA [4]. In addition, it is rich in vitamin A, E, and carotenoid astaxanthin, which makes it more stable and resistant to oxidation, compared to fish oil in terms of biological effects [5,6]. Krill oil is also associated with the regulation of lipid metabolism, inflammation, and oxidative stress [7].

Given this perspective, considering the nutritional quality of krill oil and its benefits, it is questioned whether it has any influence on adiposity. In men with obesity, consumption of krill powder for 24 weeks reduced the waist-to-hip ratio [8]. Thus, knowing that waist circumference is a diagnostic marker of excess adiposity [9] and that the sagittal abdominal diameter is an anthropometric measure positively associated with body fat and sensitive to the detection of abdominal overweight in women [10,11], evaluating the impact of krill oil on these variables in women can contribute to the treatment of overweight. Thus, our primary outcome was evaluating the effects of krill oil on the reduction of waist circumference and sagittal abdominal diameter in overweight women.

## 2. Methods

### 2.1. Trial Design

**Figure 1:**
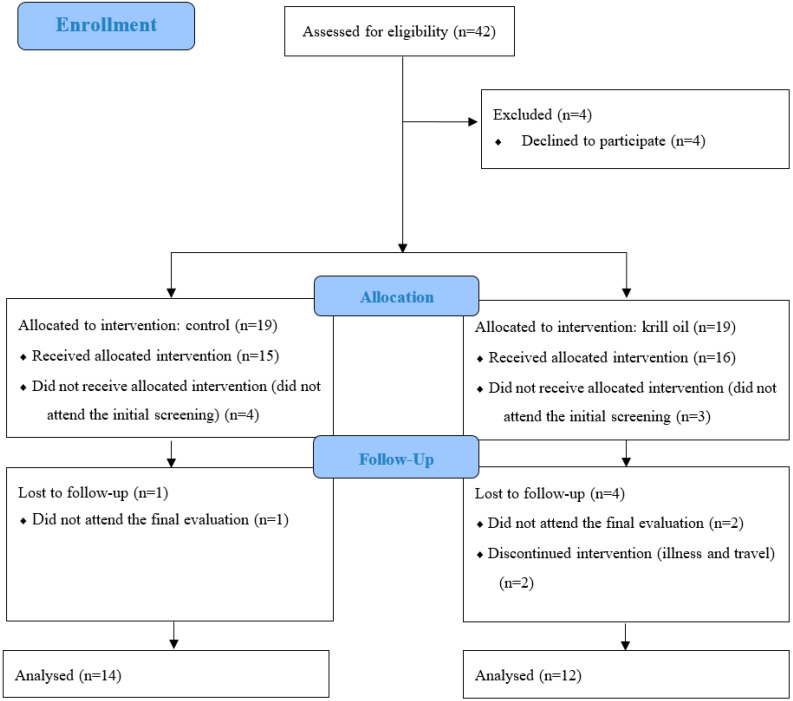
Participant flowchart.

This pilot, balanced, double-blind, parallel design, and a placebo-controlled clinical trial was conducted for eight weeks. Women were recruited through social media and 42 were eligible to participate according to inclusion criteria. Of these, 38 women were balanced according to the classification of age and body mass index (BMI), to avoid selection biases. Of these, 31 women attended the initial screening and were allocated into one of two groups: control (CG) (*n* = 15) and krill oil (GK) (*n* = 16) at the beginning of the study (Figure 1). No participants changed the group assigned at the beginning of the study.

### 2.2. Sample Calculation and Participants

A sample calculation was performed considering a clinical trial type study to compare the weight loss averages of 10% two-tailed type, the absolute error of 5%, the effect size of 2.25, and test power of 95%, which determined a sample of 15 individuals for each group; to which 50% were added to cover possible losses during the collection process, totaling 21 patients per group and 42 in the study. The sample calculation was performed using G*Power software version 3.2.9.2 (Franz Faul^TM^, Kiel, Germany).

Adult women were included in the study, aged between 20 and 59 years, and with a body mass index ≥25 kg/m^2^. Women diagnosed with kidney, cardiovascular, liver, and/or cancer diseases were excluded; pregnant; lactating; being chronic drinkers; using an anti-inflammatory, for loss of appetite or body mass drugs; in an inflammatory or infectious process on the day of the evaluation or the previous seven days; participating in any food restriction program or following a specific diet; using dietary supplements in the last six months; and being intolerant or allergic to seafood in general. The selection of the participants into the study was based on participant characteristics observed before the start of the study.

The participants who agreed to participate in the study signed the informed consent form, according to the National Health Council of the Brazilian Ministry of Health. This study was approved by the local research ethics committee (number: 2.509.400/CAAE: 82445318.5.0000.5083) and registered in the Brazilian Registry of Clinical Trials (ReBEC) under the number RBR-6dq7tz.

### 2.3. Supplementation Protocol

The control group (CG) (*n* = 15) received oil capsules containing 500 mg of mineral oil in each and were instructed to consume three in the morning and three in the afternoon, totaling 3 g of oil per day. The krill oil group (KG) (*n* = 16) received oil capsules containing 500 mg of krill oil (Pholias^TM^, Anápolis, Goiás) in each and were instructed to consume three in the morning and three in afternoon snacks, totaling 3 g of krill oil containing 500 mg of eicosapentaenoic acid (EPA) per day. The supplementation time in both groups was eight weeks.

**Figure 2:**
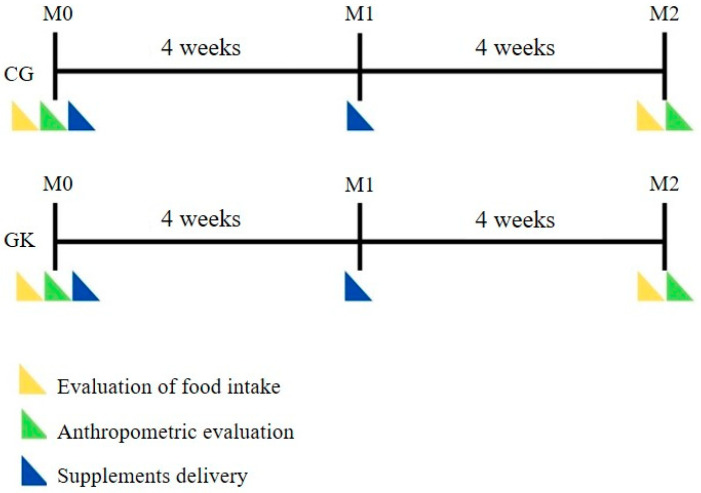
Experimental draft for both groups. M0: Initial screening; M1: Last supplements delivery; M2: Final evaluation. CG: Control group; GK: Krill oil group.

A person not involved with the study performed the blinding of the supplements by packing the mineral oil and krill oil capsules in different bottles; however, of the same size and opaque color, differentiated only by the identification tag with the name of the participants. Opaque bottles were important since the mineral oil capsules were transparent, and the krill oil capsules were dark red. The capsule bottles containing placebo or krill oil were delivered to the participants at moments (M0) and (M1) (Figure 2). In M1 and M2, the participants returned the bottles delivered previously and the remaining capsules were counted to evaluate the adhesion.

### 2.4. Data Collection

Data collection was conducted in two moments of evaluation: initial screening (M0) and final evaluation after eight weeks of supplementation (M2) (Figure 2). The measurements of body mass and height were performed according to the procedures described by Lohman et al. [12]. To obtain body mass, an adult digital anthropometric scale from Plenna Acqual, Brazil, São Paulo, with a capacity of 150 kg and precision of 100 g was used. The height value was obtained using the AVANUTRI stadiometer. The reference values used for classification were those proposed by the World Health Organization [13]. 

The body mass index (BMI) was calculated according to the body mass (kg) and height (m) data, using the formula: BMI = weight (kg)/(height)^2^. The cutoff points for adults were those recommended by the World Health Organization [13]. 

The waist circumference was determined with the aid of a measuring tape, which was placed without applying pressure, in a horizontal plane at the level of the natural waist. In cases of difficulty in identifying the natural waist, the measurement was performed at the midpoint between the lower portion of the last rib and the iliac crest [14]. 

Since the sagittal abdominal diameter is a simple anthropometric measure with a significant correlation with insulin resistance [11], an alternative was proposed to assess body fat distribution, specifically intra-abdominal fat. The participants were instructed to lie supine on a stretcher and the measurement was performed in the region of the umbilical scar, using the anthropometric wooden ruler with a movable stem. This evaluation took place in M0 and M2 (Figure 2).

Handgrip strength was used to evaluate the muscle strength in the initial (M0) and final moments (M2). The participants were instructed to sit in a chair with a straight back and without support for the arms; keeping the shoulder adducted and neutrally rotated; the elbow flexed at 90°; the position and the wrist between 0 and 30° of extension, and between 0 and 15° of ulnar deviation [15]. Subsequently, they were instructed to tighten the equipment with as much force as possible three times in each hand with an interval of one minute between measurements, being the highest measures used in the analysis.

The volunteers were submitted to the assessment of food intake at times M0 and M2 through the 24 h food recall (R24H) on three different days (two days a week and one day on the weekend), which were completed following the “USDA’s Automated Multiple-Pass Method” [16]. The data only considered food ingestion and not supplementation. The chemical analysis of food consumption was performed using the Dietpro^®^ software version 5.8 (Agromídia Software^℗^, Viçosa, Brazil).

### 2.5. Statistical Analyses 

The participants who had an intake of more than 70% of the capsules were considered in the analysis. Differences (∆) were calculated by subtracting the post- from the pre-intervention values. To assess the distribution of data, the Shapiro-Wilk test was used. The variables were described as mean and standard deviation. The t Student paired or unpaired test, Mann–Whitney, and Wilcoxon test were used to compare the groups or the times of intervention. The chi-square test was performed to assess the successes and errors of the subjects when asked which supplement they were taking. Statistical analyses were performed using the RGui (64-bit) and RStudio software, and a 5% significance level was adopted (*p* < 0.05).

## 3. Results

After eight weeks of intervention, data from 14 participants were analyzed in the control group and 12 in the krill group. In the control group, there was one loss to follow-up; in the krill group, two losses to follow-up; and two participants discontinued the intervention. Regarding dropping out of the study, there was no difference between the groups (control group 26% vs. krill group 37.8%, *p* = 0.38). In addition to that, no significant differences were found comparing both groups regarding anthropometrics and handgrip strength measurements. Right handgrip strength increased for the control group (*p* = 0.020) and krill group (*p* = 0.005), and left handgrip strength increased for the krill oil group (*p* = 0.012) (Table 1).

The food intake was different between the groups for carbohydrate in grams and kilocalories (*p* = 0.040), and for polyunsaturated fatty acids (*p* = 0.006). Considering the control group, the intake of lipids post-intervention in grams and kilocalories (*p* = 0.013), monounsaturated (*p* = 0.006) and polyunsaturated (*p* = 0.048) fatty acids decreased compared to the pre-intervention time. For the krill oil group, an increase of the intake of polyunsaturated fatty acids (*p* = 0.049) was detected, comparing pre- to post-intervention (Table 2). 

When assessing what each volunteer believed to have consumed, in CG, 42.9% believed it to be placebo, 21.4% believed it to be krill oil, and 35.7% did not know how to respond, while in GK, 66.7% believed it to be krill oil and 33.3% did not know how to respond. The *p* value for the proportion differences between error and success was *p* = 0.016.

## 4. Discussion

In the present article, the supplementation with three g/day of krill oil was neither able to change the waist circumference and the sagittal abdominal diameter, nor to modify the handgrip strength of women who were excessively overweight. The increase in carbohydrate and polyunsaturated fatty acid consumption observed in the krill oil group happened by chance, as supplementation was not included in the 24 h recall calculation and participants were asked to maintain the dietary pattern during the study. Although previous studies suggest that n-3 PUFA influences food intake and is related to metabolic changes that impact on loss of body mass [3,17], studies evaluating the interaction of these factors by the consumption of krill oil are limited and present methodologies make comparison difficult [8,18,19].

In this sense, the study of Trepanowski et al. [18] found no effects of the interaction of supplementation with krill oil (2 g/day) on anthropometric and food intake variables in individuals undergoing fasting (Daniel Fast) for 21 days. In this study, people between 19 and 65 years old (*n* = 12 men and 27 women) were recruited and divided into two groups: krill oil (two g/day) or placebo (coconut oil: two g/day), who were subjected to Daniel fasting, a diet that prohibits the consumption of animal products, refined foods, white flour, preservatives, additives, sweeteners, caffeine, and alcohol. It is noted that, despite having supplemented krill oil, the food restriction observed in the study and the short intervention period may have limited the observation of the results. In addition, it is important to emphasize that the joint analysis of individuals of both sexes and a lack of consideration of the extent of the age group may have caused a bias in the study, since we know that body composition may be influenced by sex and age [20].

In a pilot study conducted with individuals overweight and obese, no differences were found in BMI and waist circumference between the groups that received krill oil (*n* = 21), omega-3 (*n* = 23), and control (*n* = 19) [19]. It is noteworthy that in this study, there was a small participation of male individuals, which preserved statistical analysis by gender, and the inclusion of a heterogeneous sample with women in pre- and post-menopause, factors that influence the outcomes of the study.

Another pilot study carried out with men with excess body fat evaluated the effect of krill powder on lipid profile indicators. The dose of four g/day was supplemented for 24 weeks; however, there was no difference in body mass compared to the control group. However, there was a significant difference in the waist/hip ratio and the visceral fat and skeletal muscle mass ratio [8]. Such findings ask whether chronic studies could show a reduction in weight and body adiposity. As the effects of krill powder are still poorly established and because it is a pilot study that did not evaluate food intake, it is questioned whether the different forms of krill supplementation (powder and oil) can interfere with the results, especially those which refer to appetite and body mass. 

Thus, we note the absence of clinical trials evaluating the effects of krill oil on food intake, which is a positive point for our study, as although it does not indicate important results, it is a pioneering work in evaluating women who are overweight. In addition, our work indicates the need for further studies, with a larger sample size, and more time and supplementation, to establish the practical functionality of consuming krill oil in the routine of overweight individuals. We emphasized that the mineral oil and krill oil capsules with different colors are a limitation of our work, which should be considered in further studies. In addition, the way to identify treatment compliance may not have captured the intake of the supplement. Another limitation refers to the identification of which supplement the participants consumed. However, the placebo effect did not happen in this sample, even though the participants knew what they were taking.

## 5. Conclusions

In conclusion, supplementation with krill oil in overweight women did not reduce the waist circumference and sagittal abdominal diameter. However, long-term studies with a larger sample size are necessary to evaluate the possible benefits of krill oil supplementation in women with excess adipose tissue.

## Figures and Tables

**Table 1:** Anthropometric and handgrip strength measurements of participants.

Variables	Control Group (*n* = 14)			Krill Oil Group (*n* = 12)			*p* ^†^
	Pre	Post	∆	Pre	Post	∆	
Body mass (kg)	79.8 ± 12.8	79.9 ± 12.3	0.1 ± 1.6	77.1 ± 9.97	77.5 ± 11.1	0.4 ± 1.8	0.690
BMI (kg/m^2^)	31.2 ± 4.4	31.2 ± 4.2	0.0 ± 0.6	30.9 ± 3.5	30.9 ± 4.0	−0.1 ± 0.9	0.644
WC (cm)	98.4 ± 11.2	98.5 ± 11.5	0.0 ± 4.7	96.7 ± 10.0	96.7 ± 11.1	−0.1 ± 3.5	0.536
SAD (cm)	23.2 ± 3.7	22.2 ± 3.1	−1.0 ± 3.2	22.5 ± 3.0	20.8 ± 2.6	−1.6 ± 2.4	0.598
HGSr (mm)	24.9 ± 5.2	26.8 ± 4.9 ^††^	1.8 ± 2.4	23.2 ± 4.3	25.6 ± 4.3 ^††^	2.4 ± 2.0	0.543
HGSl (mm)	24.5 ± 5.4	24.8 ± 5.4	0.3 ± 2.4	22.1 ± 5.3	24.4 ± 4.9 ^††^	2.3 ± 2.6	0.051

Values are presented as mean ± standard deviation from the mean. BMI: body mass index; WC: waist circumference; SAD: sagittal abdominal diameter; HGSr: right handgrip strength, HGSl: left handgrip strength. ^†^ Obtained by the analysis of t Student unpaired test or Mann–Whitney, comparing differences (∆) between groups. ^††^ Differences from pre-intervention (*p* < 0.05) obtained using t Student paired test or Wilcoxon test.

**Table 2:** Total food consumption of the participants.

Variables	Control Group (*n* = 14)			Krill Oil Group (*n* = 12)			*p* ^†^
	Pre	Post	∆	Pre	Post	∆	
Energy (kcal)	1744.2 ± 383.0	1584.2 ± 455.1	−160.0 ± 449.5	1311.5 ± 523.1	1448.8 ± 455.4	137.3 ± 381.3	0.080
Carbohydrate (g)	220.5 ± 63.0	186.4 ± 50.5	−34.1 ± 62.6	158.5 ± 65.6	174.2 ± 54.5	15.7 ± 38.5	0.040 *
Carbohydrate (%)	50.3 ± 8.9	52.2 ± 7.0	1.9 ± 8.6	49.0 ± 6.7	52.9 ± 4.0	4.0 ± 9.0	0.719
Carbohydrate (kcal)	882.3 ± 252.0	745.8 ± 202.2	−136.4 ± 250.5	634.14 ± 262.7	696.9 ± 218.1	62.8 ± 154.0	0.040 *
Proteins (g)	69.5 ± 17.9	58.9 ± 23.1	−10.6 ± 19.8	57.25 ± 26.3	50.5 ± 20.1	−6.7 ± 26.9	1.000
Proteins (%)	16.0 ± 3.2	16.5 ± 3.0	0.5 ± 4.3	16.8 ± 5.4	15.0 ± 1.9	−1.8 ± 5.6	0.181
Proteins (kcal)	278.0 ± 71.8	235.8 ± 92.5	−42.3 ± 79.3	229.0 ± 105	202.2 ± 80.6	−26.8 ± 107.5	1.000
Proteins (g/kg)	0.8 ± 0.2	0.7 ± 0.3	−0.1 ± 0.2	0.7 ± 0.3	0.6 ± 0.2	−0.1 ± 0.3	0.959
Lipids (g)	62.0 ± 18.8	50.3 ± 18.8 ^††^	−11.6 ± 14.2	48.0 ± 19.4	48.6 ± 21.7	−0.6 ± 16.6	0.064
Lipids (%)	33.1 ± 5.7	30.2 ± 5.6	−2.9 ± 6.4	32.7 ± 3.9	31.8 ± 3.9	−0.9 ± 3.1	0.382
Lipids (kcal)	558.0 ± 169.3	453.4 ± 169.8 ^††^	−104.5 ± 128.1	432.1 ± 175.4	438.0 ± 195.9	5.9 ± 149.1	0.064
SFA (g)	19.4 ± 5.2	16.0 ± 7.4	−3.4 ± 6.5	15.3 ± 7.7	11.9 ± 5.8	−3.4 ± 8.5	0.877
MFA (g)	18.6 ± 5.2	13.9 ± 6.0 ^††^	−4.7 ± 4.6	14.3 ± 7.4	13.1 ± 8.2	−1.2 ± 7.9	0.217
PFA (g)	7.5 ± 3.6	5.3 ± 3.2 ^††^	−2.2 ± 3.4	4.9 ± 3.6	6.9 ± 4.4 ^††^	2.0 ± 3.0	0.006 *

Values are presented as mean ± standard deviation from the mean. SFA: saturated fatty acids; MFA: monounsaturated fatty acids; PFA: polyunsaturated fatty acids. ^†^ Obtained by the analysis of t Student test or Mann–Whitney comparing differences (∆) between groups. * *p* < 0.05 was considered with significant difference. ^††^ Differences from pre-intervention (*p* < 0.05) obtained using t Student paired test or Wilcoxon test.
